# Internal pH and Acid Anion Accumulation in *Listeria monocytogenes* and *Escherichia coli* Exposed to Lactic or Acetic Acids at Mildly Acidic pH

**DOI:** 10.3389/fmicb.2021.803271

**Published:** 2022-02-24

**Authors:** Savannah R. Branson, Jeff R. Broadbent, Charles E. Carpenter

**Affiliations:** Department of Nutrition, Dietetics and Food Sciences, Utah State University, Logan, UT, United States

**Keywords:** organic acid, *Listeria monocytogenes*, *Escherichia coli*, anion accumulation, intracellular pH

## Abstract

Organic acids are widely employed in the food industry to control growth of microbial pathogens such as *Listeria monocytogenes* and *Escherichia coli.* There is substantial evidence that intracellular accumulation of acid anions is a major inhibitor to cell viability, and that some bacteria are able to combat the toxic effects of anion accumulation *via* their ability to continue active metabolism at a lower intracellular pH (pH_i_). This study followed the accumulation of acid anion into the cell pellet and parallel changes in pH_i_ in two human pathogenic strains of *L. monocytogenes* (N1-227 and R2-499) and in *E. coli* O157:H7 after exposure to sub-bacteriostatic levels of lactic and acetic acids at mildly acidic pH 6. The methodology employed in these studies included independent measures of pH_i_ and intracellular anion accumulation. For the latter work, cells were pelleted through bromododecane to strip off extracellular water and solutes. *Listeria* strains accumulated 1.5-fold acetate or 2.5-fold lactate as compared to the external environment while mounting a defense against anion accumulation that included up to a 1-unit pH_i_ drop from 7.5 to 6.5 for strain R2-499. *E. coli* accumulated 2.5-fold acetate but not lactate and apparently made use of combat mechanisms other than lowering pH_i_ not explored in this study. Inulin was employed to estimate the fractional volume of cell pellet present as intracellular space. That intracellular fraction was 0.24 for *E. coli*, which infers that acid accumulation into the intercellular space was minimally 4 × that measured for the entire pellet. An intercellular fraction of pellet was not measurable for strains of *L. monocytogenes*. The data also bring into question the efficacy across bacterial species of the common, but confounding, practice of using intracellular anion accumulation as a measure of pH_i_, and *vice versa*.

## Introduction

*Listeria monocytogenes* and *Escherichia coli* are pathogens of concern for foodborne illness, and each is among the top five causes of food-related death (Mead et al., 1999; Scallan et al., 2011). Both pathogens are hardy organisms that can survive in many different types of environments, including food production facilities (LeJeune et al., 2001; Popovic et al., 2014). The food industry often uses organic acids in the environment and as food additives to limit growth of these and other pathogens. For example, acetic and lactic acids are used to decontaminate meat surfaces of *E. coli* and *L. monocytogenes* (Berry and Cutter, 2000) and as antilisterial additive in meat products (Dziezak, 2016).

It is hypothesized that intracellular accumulation of acid anions is a primary mechanism by which organic acids exert antimicrobial activity (Mani-López et al., 2012), and that such accumulation is driven by the difference in external and internal pH (Carpenter and Broadbent, 2009). It is also understood that some bacteria can lower their internal pH (pH_i_) as a primary mechanism to combat accumulation of organic acid anions (Russell, 1992; Diez-Gonzalez and Russell, 1997). Other researchers have focused on the external protonated acid driving internalization of acid anion (Buchanan et al., 1994; Romick and Fleming, 1998; Pieterse et al., 2005). In contrast, Carpenter and Broadbent (2009) assert in their hypothesis paper that external protonated acid is merely a shuttle, not a driving force for intracellular anion accumulation. Their hypothesis paper points out that a failure to control external anion concentration has confounded results and likely led to misleading conclusions regarding the antimicrobial action of organic acids.

Hence, this study explored the extent to which mid-log phase cells of two human pathogenic strains of *L. monocytogenes* and one *E. coli* O157:H7 strain accumulate acid anions and respond with altered pH_i_ when exposed to inhibitory, but less-than-bacteriostatic, levels of organic acids at mildly acidic pH 6 typical of mildly acidic foods such as processed meats. External concentration of organic acids was set at 4.75 mM for both lactic and acetic acid consistent with our previous studies using the two *Listeria* strains (Zhang et al., 2014; Zhang et al., 2015; Li et al., 2021). *E. coli* O157:H7, a Gram-negative and broadly studied human pathogen, was included to provide a basis for comparison.

## Materials and Methods

### Experimental Overview and Statistical Analysis

Experiments were conducted to follow the extent to which added organic acids induced relative accumulation of organic acid anions into the cell pellet and to identify the fractional volume of cell pellet not available to inulin (i.e., intracellular space). Parallel experiments were conducted to follow the impact of treatments on internal pH of the bacterial cells. Independent experiments were performed using two human pathogenic strains of *L. monocytogenes* and *E. coli* O157:H7. Each experiment included four trials, with three replications per trial.

For the acid accumulation experiments, treatment levels included TSB media containing 4.75 mM added lactic or acetic acid and adjusted to pH 6 with HCl. The dependent measure was a relative accumulation factor calculated as:


$$\left(\frac{C14pelletcounts}{H3pelletcounts}\right)/\left(\frac{C14supernatantcounts}{H3supernatantcounts}\right)$$

The accumulation factor was considered significant when its 95% CI > 1. Treatment means were compared using one-way analysis of variance (ANOVA), and differences were identified by Tukey’s test at *p* < 0.05.

For the inulin experiments, one treatment level (standard TSB adjusted to pH 6 with HCl) was applied to identify the fraction of the pellet volume unavailable to inulin (i.e., a fractional internal cell volume). This fractional volume was calculated as the C^14^:H^3^ ratio of pellet as compared to the C^14^:H^3^ ratio of supernatant. For example, a C^14^:H^3^ ratio in pellet that was only 80% of the C^14^:H^3^ ratio in the supernatant corresponds to a fractional internal cell volume of 0.2. Calculation of the fractional internal cell volume was only warranted if the C^14^:H^3^ ratio in the pellet was lower than the C^14^:H^3^ ratio in the supernatant (*p* < 0.05, *t*-test).

Four treatments were included in the pH_i_ experiments: the baseline control (TSB pH 7.4), acid control (TSB adjusted to pH 6 with HCl), and TSB with 4.75 mM added lactic acid or acetic acid and adjusted to pH 6 with HCl. The dependent variable was the pH_i_ measured using a pH-sensitive fluorescent dye as described later. Treatment means were compared using one-way analysis of variance (ANOVA), and differences were identified by Tukey’s test at *p* < 0.05.

### Bacterial Strains and Growth Conditions

Two strains of *L. monocytogenes* were selected for this study, FSL R2-499 and FLS N1-227. The R2-499 strain was sourced from a human isolate associated with a United States outbreak linked to sliced turkey and the N1-227 strain was sourced from a food isolate associated with a United States outbreak (Li et al., 2021). One strain of *E. coli* O157:H7 was selected, H1730. This strain was sourced from a human isolate associated with a lettuce outbreak (Jensen et al., 2015).

Parent cultures were stored as frozen stocks at −80°C in tryptic soy broth (TSB, pH 7.4; Becton, Dickinson and Company, Sparks, MD, United States) supplemented with 20% v/v glycerol. Prior to use, cultures were first propagated on tryptic soy agar (TSA; Becton, Dickinson and Company, Sparks, MD, United States) plate and incubated at 37°C for 24 h. A single colony from the TSA plate was transferred into TSB and incubated overnight at 37°C with shaking (220 rpm).

### Accumulation of Organic Acid and Inulin Into Cell Pellets

Bacterial cells habituated to treatment media containing added organic acids are expected to proportionately accumulate acid anions into their cytoplasm relative to the supernatant owing to the protonated acid freely entering the cell but then dissociating into a proton and acid anion. On the other hand, bacterial cells habituated to treatment media containing inulin are expected to have proportionally less inulin in the pellet (which holds the cytoplasmic fraction) relative to the supernatant owing to inulin not being able to enter the cell. The proportionate amounts of organic acids and inulin was followed using their C^14^ radio-labeled tracers, and a tracer of tritium was simultaneously employed because it will freely equilibrate across all cell compartments and the media. Hence, the ratio of C^14^:H^3^ indicated relative concentration of the organic acids or inulin in pellet and in supernatant.

Overnight cultures of each strain were harvested by centrifugation (2,500 × *g* for 10 min; Sorvall RT1, Thermo Fisher Scientific, Germany) at 4°C, and then diluted to an optical density at 600 nm (OD_600_) of 0.03 in TSB. Cells were acid habituated as described by Zhang et al. (2014). A 1% inoculum (v/v) of diluted overnight cultures was transferred into 10 ml of standard TSB (pH 7.4) and incubated at 37°C for 3 h for *E. coli* and 4 h for *L. monocytogenes* with shaking (220 rpm) to reach mid-log phase. Cell density was approximately 10^7^ CFU/ml (Zhang et al., 2014). The cultures were collected by centrifugation (2,500 × *g* for 10 min at 4°C) and then resuspended in 10 ml of a treatment solution made from standard TSB (pH 6.0 adjusted with HCl, Becton, Dickinson and Company, Sparks, MD, United States). The two acid treatments contained 10 μCi tritiated water (Perkin Elmer, Waltham, MA, United States) and either 4.75 mM acetic acid (Johnson Matthey Company, Ward Hill, MA, United States) containing 1 μCi C^14^-labeled acetic acid (Perkin Elmer, Waltham, MA, United States), or 4.75 mM L-lactic acid (Sigma Chemicals, St. Louis, MO, United States) containing 1 μCi C^14^-labeled lactic acid (Perkin Elmer, Waltham, MA, United States). The inulin treatment contained 10 μCi tritiated water and 1 μCi C^14^-labeled inulin (Perkin Elmer, Waltham, MA, United States).

Cells were incubated for 1 h at 37°C with shaking (220 rpm). After incubation, 0.5 ml of bromododecane (Sigma Aldrich, St. Louis, MO, United States) was added to the centrifuge tube to create a layer between the supernatant and pellet after centrifugation. Cells were collected by centrifugation (2,500 × *g* for 10 min at 4°C) and then the supernatant was decanted and diluted so that disintegrations per minute (DPM) would be similar to that of the pellet. One milliliter of the diluted supernatant was added to 18 ml of liquid scintillation cocktail (Perkin Elmer, Waltham, MA, United States). The entire cell pellet was cut out of the centrifuge tube using guillotine clippers and then added to 18 ml of liquid scintillation cocktail along with 1 ml of TSB. Liquid volume in both the supernatant and pellet samples was identical (1 ml) to ensure similar counting efficiency. Counts were measured using a scintillation counter (Beckman Coulter, Brea, CA, United States).

### Measurement of pH_i_

The pH_i_ was determined using 5(6)-carboxyfluorescein diacetate N-succinimidyl ester (CFSE; Sigma Aldrich, St. Louis, MO, United States), a membrane-permeant fluorescein-based dye that at 492 nm is sensitive to pH (Siegumfeldt et al., 1999). This procedure generally followed that described by Siegumfeldt et al. (1999) and Cheng et al. (2015). The first step was to determine a calibration curve that would relate fluorescent values to pH_i_ values. Ten milliliters of mid log cells grown in TSB were collected by centrifugation and resuspended in 10 ml of CFSE staining solution (10 μM; prepared from a concentrated stock solution in DMSO by dilution in a 10 mM KH_2_PO_4_ buffer) and incubated at 37°C for 30 min. The cells were collected by centrifugation and then suspended in 10 ml of KH_2_PO_4_ buffer (pH 6.0) supplemented with 10 mM glucose (to energize the cells) and incubated at 37°C for another 30 min to remove unbound dye. To permeabilize cells and equilibrate intracellular and external pH for the calibration curve, the stained cells were then placed in ethanol (63%, v/v) for 30 min at 37°C. The bacterial cells were harvested by centrifugation (2,500 × *g* for 10 min at 4°C) and suspended in 10 ml of TSB broth medium whose pH was adjusted with HCl or NaOH to range from 5.0 to 8.0 in 0.5 increments. Fluorescence was measured using a microplate fluorometric reader (BioTek, Winooski, VT, United States). The 492/435 fluorescent ratio was obtained by dividing fluorescence at 492 nm by that at 435 nm. These wavelengths are pH sensitive and pH insensitive, respectively. Undyed cells in TSB were used as a blank to correct for any naturally occurring fluorescence. The calibration curve was plotted by polynomial fitting between ratio 492/435 and the pH_i_ of the equilibrated cells corresponding to the broth pH (5.0 to 8.0), respectively. It was necessary to establish a different calibration curve for each strain because of the nature of the intracellular dye binding to molecules within the cell that could vary by strain. After the curve was established, polynomial fitting was used to establish an equation for each strain relating fluorescence values (492/435 nm) to pH_i_.

Mid log cells (cell density approximately 10^7^ CFU/ml) were exposed to one of four treatments described in the experimental overview and as described by Li et al. (2021). Cells were incubated at 37°C with shaking in the microplate fluorometric reader for 60 min. Fluorescence values were measured at 492 and 435 nm, and the pH_i_ was calculated from the mean value plotted against the pH calibration curve.

## Results and Discussion

### Accumulation of Acid Anions

**FIGURE 1:**
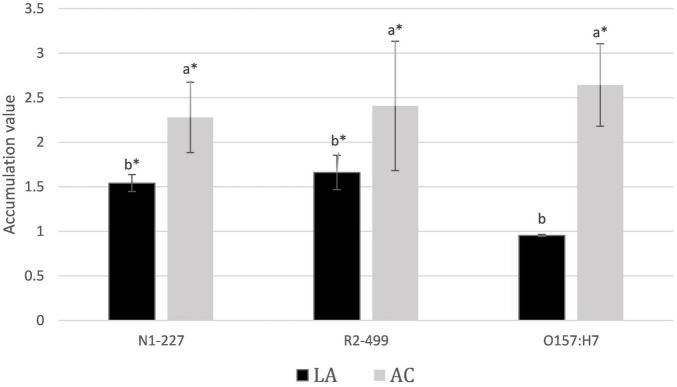
Average relative acid anion accumulation after 1 h. Relative acid anion accumulation into pellets of *Listeria monocytogenes* N1-227, *L. monocytogenes* R2-499, and *Escherichia coli* O157:H7 following 1 h in habituation media. Values represent treatment means and include bars of the standard error. Treatment details: LA, lactic acid treatment (TSB adjusted to pH 6.0 with HCl containing 4.75 mM lactic acid); AC, acetic acid treatment (TSB adjusted to pH 6.0 with HCl containing 4.75 mM acetic acid). Superscripts identify significant differences within strains (*p* < 0.05). Asterisk signifies that accumulation was significantly greater than 1 (CI < 95%).

Results from acid accumulation studies are presented in Figure 1. Lactate accumulated 1.5- and 1.7-fold in the pellets of *Listeria* N1-227 and R2-499, respectively, as compared to levels found in the supernatants. No significant accumulation of lactate was detected in the *E. coli* pellet. In comparison, the accumulation of acetate into the cell pellet was 2.3-, 2.4-, and 2.6-fold greater than in the supernatant for all *L. monocytogenes* N1-227, R2-499, and *E. coli* O157:H7, respectively. Overall, all three strains accumulated acetate anion into the cell pellet as compared to the supernatant to a greater extent than they accumulated lactic acid.

Prior studies report that acetic acid inhibits growth of *L. monocytogenes* more than lactic acid in terms of total acid added as weight by volume (Farber et al., 1989; Sorrells et al., 1989; George et al., 1996), and the results presented here suggest that this may be due to the greater relative accumulation of acetate into the cell. Roe et al. (1998) reported that at pH 6, growth in 8 mM acetate resulted in an internal pool of 240 mM acetate anion in *E. coli* representing a 30-fold relative accumulation of acetate. Results here were much lower in comparison. Thus, C^14^ inulin was employed as a control to try and assess how much of the pellet volume was truly intracellular. The fractional volume of the pellet not available to inulin as compared to water was calculated as representative of the relative intracellular space. A fractional volume of 0.24 was identified for *E. coli*, suggesting that intracellular accumulation of anion is possibly fourfold greater than measured in the pellet. Both *L. monocytogenes* strains did not present a fractional volume significantly different from zero, suggesting that it was below the detection limit of our procedure. While the methodology employed here was able to identify significant accumulation of organic acids into the pellet, our results with inulin suggest that the pellet values underestimate intracellular concentrations of anion, and the true extent of intracellular anion accumulation is likely much higher than measured for the pellet.

One possible explanation for not finding a significant fractional internal volume in *Listeria* may be related to past observations that the OD_600_ for *Listeria* strains does not correlate well with actual viable cell numbers (Zhang et al., 2014). The reason for this discrepancy is unknown, but we hypothesize that it could be due to cell clumping. If this hypothesis is correct, clumping could result in co-precipitation of media components with the pellet (despite centrifugation through bromododecane) and thereby obscure measures specific to the cell itself. Another consideration is that Gram-negative bacteria like *E. coli* may experience greater carry-along due to their periplasm that serves as a multipurpose compartment separate from the cytoplasm (Miller and Salama, 2018) and possibly having a different pH than the cytoplasm (Lund et al., 2014).

### Intracellular pH

**FIGURE 2:**
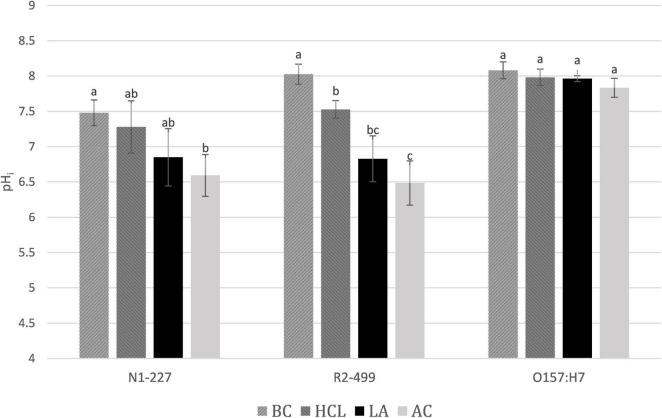
Average pH_i_ after 1 h. pH_i_ values of *Listeria monocytogenes* N1-227, *L. monocytogenes* R2-499, and *Escherichia coli* O157:H7 after 1 h in habituation treatment media. Error bars represent standard error of the mean. Treatment details: BC, baseline control (TSB pH 7.4); HCl, pH control (TSB adjusted to pH 6.0 with HCl); LA, lactic acid treatment (TSB adjusted to pH 6.0 with HCl containing 4.75 mM lactic acid); AC, acetic acid treatment (TSB adjusted to pH 6.0 with HCl containing 4.75 mM acetic acid). Superscripts identify significant differences within strains.

Results from pH_i_ studies are presented in Figure 2. Habituation to the acid control induced lowered pH_i_ in R2-499 as compared to baseline, and the trend was in that direction for N1-277. Habituation to acetic acid resulted in a significantly lower (*p* < 0.05) pH_i_ compared to exposure to the baseline control or to the acid control in both *Listeria* species. Additionally, the acid control and lactic acid were intermediate (though not necessarily significantly different) from the baseline control or acetic acid in both *Listeria* species. While the extent of the response was strain- and acid-dependent, it appears that both *Listeria* strains tolerate a lower pH_i_ to combat the toxic impacts of either external acidity or the presence of organic acids. The ability to continue active metabolism at lower pH_i_ is an important characteristic contributing to the capacity of some bacteria to resist the toxicity of organic acids (Russell, 1992; Diez-Gonzalez and Russell, 1997). It has not been previously reported that *Listeria* develops a lower pH_i_ in response to just external acidity (Budde and Jakobsen, 2000; Shabala et al., 2002), although this finding has been reported in lactic acid bacteria (Siegumfeldt et al., 2000). Regarding acid type, both lactate and acetate are part of normal metabolism in *L. monocytogenes* (Kelly and Patchett, 1996; Wallace et al., 2017), and strains may have alternative methods and varying capacity to export lactate and acetate. It is likely that the cell only develops a lowered cell pH_i_ once the rate of anion export becomes overwhelmed by passive uptake of acid, and acetic acid is significantly more lipophilic than lactic acid.

In contrast to what was observed for *Listeria*, pH_i_ of *E. coli* did not significantly change in response to any of the four treatments. However, the pH_i_ was measured after 60 min of habituation, which would miss the transient change in pH_i_ reported by Martinez et al. (2012). The near-neutral pH_i_ of *E. coli* predicts that acid anions will accumulate to a much greater extent than for the *Listeria* strains. Yet this research found that *E. coli* accumulated acetate at a level that was comparable to both *Listeria* strains and accumulated even less lactate than either *Listeria* strain. We speculate that *E. coli* adapts over 60 min to combat the accumulation of acid anions by mechanisms that are not available to the *Listeria* strains used in this work. For example, *E. coli* may change their membrane composition or upregulate export mechanisms (Kelly and Patchett, 1996; De Jonge et al., 2003).

## Conclusion and Future Research

This study used two parallel experiments to investigate how *L. monocytogenes* and *E. coli* O157:H7 respond to organic acids in mildly acidic pH environments. The two *Listeria* strains have been studied previously in our laboratory under similar conditions, thereby building on a long history with these two strains, while *E. coli* was used as an outside “control” and has been studied by other researchers. It is recognized that the use of only two strains of *L. monocytogenes* does not establish any characteristic that should be viewed as universally shared among all strains. The use of two strains has, however, allowed for more in-depth study of various phenomena and cellular mechanisms of great potential impact for food safety.

It is also worthwhile to note that these results bring into question the risks of using intracellular anion accumulation as a measure of pH_i_ as done in previous studies (Kroll and Booth, 1981; Roe et al., 1998). The methodology employed in these studies was based on independent measures of pH_i_ and intracellular anion accumulation, and the results here bring into question the common, but confounding, practice of using intracellular anion accumulation as a measure of pH_i_, and *vice versa*. Prior studies employed the common approach of utilizing C^14^-labeled organic acid to follow intracellular anion accumulation and subsequently enter the measured intracellular anion concentration along with extracellular pH into the Henderson-Hasselbach equation to predict pH_i_ (Kroll and Booth, 1981; Roe et al., 1998). Calculation of intracellular concentration of acid anion required drying of the cell pellet and calculation based on a previously established relationship between dry cell weight and internal cell volume. The risks of doing so include differences in how various species may handle acid exposure and accumulation of anions plus differences in physiology especially of Gram-positive vs. Gram negative bacteria that may interact with specific experimental protocols. This research instead used ratios of C^14^:H^3^ in the pellet and supernatant to determine the relative intracellular accumulation of acid anion in comparison to the external environment. Furthermore, a pH-sensitive fluorescent dye was employed as an independent means of measuring pH_i_.

Future research should track changes in intracellular anion accumulation and pH_i_ over time and in response to various external pH to better understand their interaction. Future studies should additionally examine the role of other mechanisms in combating the intracellular accumulation of anions including anion-specific export systems or non-specific systems such as membrane changes to combat anion accumulation (De Jonge et al., 2003; Guan and Liu, 2020). Finally, our lab has previously studied transcriptomic data of the two *Listeria* strains exposed to these conditions, and it is being considered to reexamine that transcriptomic data in connection with these results. Results from this study bring to the forefront the potential of bacteria to survive and adapt to organic acid exposure through various mechanisms including lowering pH_i_ to combat intracellular anion accumulation. While *Listeria* may lower pH_i_ as a primary mechanism to combat anion accumulation, *E. coli* may, in the long term, largely rely on means other than lower pH_i_ to combat the accumulation of anion. Additional research should be done at different external pH and external acid concentrations to truly understand the cellular response to the organic acids, and more strains will need to be studied to make broader conclusions.

## Data Availability Statement

The original contributions presented in the study are included in the article/supplementary material, further inquiries can be directed to the corresponding author.

## Author Contributions

CC and JB: conceptualization, data curation, funding, acquisition, project administration, supervision, validation, and writing—review and editing. SB: formal analysis, investigation, methodology, data collection, software, visualization, and writing—original draft. All authors contributed to the article and approved the submitted version.

## Conflict of Interest

The authors declare that the research was conducted in the absence of any commercial or financial relationships that could be construed as a potential conflict of interest.

## Publisher’s Note

All claims expressed in this article are solely those of the authors and do not necessarily represent those of their affiliated organizations, or those of the publisher, the editors and the reviewers. Any product that may be evaluated in this article, or claim that may be made by its manufacturer, is not guaranteed or endorsed by the publisher.
